# 1101. Case Study. High-voltage Electrical Groin Burn with Femoral Artery Injury: Emergent Transobturator Bypass and Burn Surgical Considerations

**DOI:** 10.1093/jbcr/irag033.205

**Published:** 2026-04-06

**Authors:** Crystal C Ukachukwu, Alan Pang

**Affiliations:** Texas Tech University Health Sciences Center, Lubbock, Lubbock, TX; Texas Tech University Health Sciences Center, Lubbock, Lubbock, TX

## Abstract

**Patient Presentation (age range, injury details, relevant history):**

A 65-year-old male was transferred to our facility after sustaining high-voltage electrical injuries involving the right groin, genitalia, torso, and bilateral lower extremities. The patient sustained 44% total body surface area (TBSA) full-thickness (third-degree) burns to the right groin (with exposed muscle), torso, and both lower extremities. Additionally, he sustained fourth-degree burns to the genitalia, resulting in complete necrosis of the penis and scrotum, and to the left foot.

**Clinical Challenges:**

On hospital day 2, the patient developed acute hemorrhage due to a blowout of the right proximal superficial femoral artery (SFA). Emergent surgical exploration revealed an approximately 2 cm defect in the proximal SFA with active pulsatile bleeding. A transobturator right external iliac artery to distal SFA bypass was performed using an 8 mm ringed, non-biologic graft. Distal perfusion to the right lower extremity was successfully restored.

**Management Approach:**

In the following days, the exposed femoral vessels were covered with a pedicled rectus femoris muscle flap. The patient underwent multiple additional reconstructive procedures to address the extent of his injuries, including:

• Allografting and autografting of burn wounds.

• Pedicled gracilis muscle flap for soft tissue reconstruction.

• Total penectomy.

• Anterior penile urethrectomy with complete bulbar urethral closure.

• Bilateral spermatic cord resection.

• Guillotine transmetatarsal amputation (TMA) of the left foot.

**Outcomes:**

Following the index revascularization and multiple soft tissue reconstruction procedures, the patient made remarkable improvement from a burn/wound standpoint. Unfortunately, he passed on from Acute respiratory failure after a 3.5 month hospital stay.

**Lessons Learned:**

Vascular injuries following high-voltage electrical trauma can evolve over time due to mechanisms such as electroporation. When electrical injury occurs in proximity to major vessels, a high index of suspicion for delayed vascular injury and necrosis is essential. These injuries often present suddenly and unpredictably, requiring continuous monitoring and preparedness for immediate operative intervention.

In contaminated fields, the transobturator bypass provides a viable revascularization option that reduces the risk of graft infection. Early involvement of vascular surgery and interdisciplinary planning is critical for limb salvage and survival in such complex burn and trauma cases.

**Applicability to Practice**:

